# Taking Down the Primary Cilium: Pathways for Disassembly in Differentiating Cells

**DOI:** 10.1002/bies.70060

**Published:** 2025-09-01

**Authors:** Carolyn M. Ott, Saikat Mukhopadhyay

**Affiliations:** 1 Janelia Research Campus, Howard Hughes Medical Institute, Ashburn, Virginia, USA; 2 Department of Cell Biology, University of Texas Southwestern Medical Center, Dallas, Texas, USA

## Abstract

Primary cilia are customized subcellular signaling compartments leveraged to detect signals in diverse physiological contexts. Although prevalent throughout mammalian tissues, primary cilia are not universal. Many non-ciliated cells derive from developmental lineages that include ciliated progenitors; however, little is known about how primary cilia are lost as cells differentiate. Here, we examine how ciliated and non-ciliated states emerge during development and are actively maintained. We highlight several pathways for primary cilia loss, including cilia resorption in pre-mitotic cells, cilia deconstruction in postmitotic cells, cilia shortening via remodeling, and cilia disassembly preceding multiciliogenesis. Lack of ciliogenesis is known to decrease primary cilia frequency and cause ciliopathies. Failure to maintain cilia can also cause primary cilia to be absent. Conversely, defects in primary cilia suppression or disassembly can lead to the presence of primary cilia in non-ciliated cells. We examine how changes in ciliation states could contribute to tumorigenesis and neurodegeneration.

## 1 ∣ Introduction

Primary cilia are microtubule-scaffolded membrane extensions customized to perceive the extracellular space [1]. Different receptors can selectively populate the ciliary membrane in different cells [1, 2]. This endows primary cilia with great versatility. Specialized sensory primary cilia are customized to detect light and odors [3]. During development, primary cilia detect chemical and mechanical signals that determine left-right asymmetry, promote morphogenetic patterning in diverse tissues, and regulate regenerative programs [4, 5]. Primary cilia-mediated signaling also influences appetite regulation and adipose tissue expansion [6]. Because ciliated and non-ciliated cells co-exist in the same tissues, the presence of a cilium enables responses to signals that are undetected by non-ciliated cells.

In studies of lung multiciliated cells, Sorokin first described a transient, solitary cilium that emerges prior to the formation of multiple motile cilia, which he named the “primary cilium” [7]. Primary cilia are typically solitary and non-beating structures found on diverse cells (not just differentiating multiciliated cells). At the primary cilium base, microtubule extend from nine centriolar microtubules, without the central doublet found in motile cilia. The transition zone above the centriole contains protein complexes linking microtubules and membrane, creating a selective barrier and enabling specialization of cilia composition [8]. Intraflagellar transport (IFT) machinery, composed of multimeric IFT complexes and specialized motor proteins, mediates the bidirectional movement of ciliary proteins across the transition zone and within the cilium, a process critical for cilium assembly, maintenance, and signaling [9].

Extensive research has focused on the presence of primary cilia: how they are built, how they function, and the pathologies that result from cilia deficiencies [6, 10]. Less attention has been given to the non-ciliated cells. Most research on primary cilia loss centers on disassembly before mitosis [11-13]. One reason that primary cilia disassembly may not have been extensively studied in non-dividing cells could be that there have been several claims that cilia are nearly ubiquitous [14]. Before their essential roles outside of sensory cells were recognized, primary cilia frequency was used as a proxy for importance—that is, prevalent structures were presumed to be functionally significant. Since the 1960s, lists of ciliated cells have documented the pervasiveness of primary cilia across tissues and species [15, 16]. Early studies used electron microscopy (EM), and finding a cilium was the fruit of persistence or luck because cilia are visible in just a few of the many sections for each cell. Primary cilia absence was noted in comparatively fewer cells, including blood cells, adipocytes, hepatocytes, and striated muscle cells [16]. Because the primary cilium was not yet considered a standard cellular component, evidence of prevalence was used to argue that cilia should be included in the definition of a cell [16]. Primary cilia were viewed by many outside the field as vestigial until the late 1990’s when evidence revealed that loss of primary cilia led to abnormalities during embryogenesis [17, 18]. Twenty-five years later, we are still learning about the many ways primary cilia influence development, physiology, and disease, but the significance of non-ciliated cells remains underappreciated.

We are well-positioned to re-examine our paradigms of primary cilia disassembly and the emergence of non-ciliated cells. Beyond cultured, cycling cells, we can now study disassembly processes within tissue and organ systems. Here, we examine the prevalence of non-ciliated cells, then explore the emergence of both ciliated and non-ciliated cells during development. We contend that there is no default ciliation state; the presence of primary cilia is either suppressed or maintained. Because cilia disassembly drives cells into a non-ciliated state, we highlight several pathways leading to primary cilia loss. Finally, we consider how studying mutations and deficiencies that alter primary cilia maintenance and primary cilia loss might provide new insights into disease pathologies.

## 2 ∣ Non-Ciliated Cells Are Diverse and Abundant

Although often overlooked, non-ciliated cells are prevalent across a wide range of tissues, as illustrated in Table 1. The absence of primary cilia is typically determined by the lack of immunostaining for cilia-specific markers or confirmed through EM. Although the list of non-ciliated cell types is extensive, it is likely incomplete for two key reasons. First, primary cilium absence has not always been reported because it was not historically viewed as biologically significant. Second, whereas single-cell RNA sequencing has greatly expanded our knowledge of cell type diversity, no comprehensive analysis of ciliation status across cell atlases has been conducted. The long-standing assumption that most cells are ciliated is challenged not only by the diversity of non-ciliated cell types but also by their abundance within the body. For example, the skin, our largest organ, is primarily composed of non-ciliated keratinocytes [19, 20]. Muscle tissue, which can account for nearly half of body mass, consists largely of myofibrils that are non-ciliated starting from the myotube stage [21]. Even within the brain, an estimated 80% of neurons are non-ciliated cerebellar granule cells (GC) [22, 23].

In Table 1, we list cells that are predominantly non-ciliated; however, the classification of ciliated or non-ciliated cell types can be imprecise because primary cilia penetrance can vary. For example, 85% of pancreatic beta cells are ciliated cells, leaving 15% that lack primary cilia [45]. Ciliation of cortical excitatory neurons is nearly 100% [24]; however, more than 20% of parvalbumin and somatostatin interneurons are non-ciliated [46]. In some tissues, cilia frequency may be less than 10% [47, 37]. Primary cilia frequency can also change within a population due to physiology [48] or disease [49]. Although in this review, we largely consider changes in ciliation state during differentiation, the mechanisms discussed could be important to generate subpopulations of non-ciliated cells.

## 3 ∣ Non-Ciliated Cells Emerge as Cells Differentiate

Primary cilia play essential roles in early development. Although embryos lacking primary cilia-forming genes can develop through the earliest stages of gestation, they typically arrest by embryonic day 9 (E9.0) in mice [17, 18, 50, 51]. This suggests that primary cilia are not required for implantation but become critical soon after. Indeed, primary cilia cannot form in the early embryo because centrosomes are immature [52, 53]. Primary cilia first appear during epiblast cavitation around embryonic day 5.5 (E5.5) in mice [33]. Nodal cilia, which drive left–right patterning, are among the earliest known functional cilia and are observed as early as E7.5 in mice [54]. By E8.0 in mice, primary cilia in non-cycling cells are pervasive [33]. In contrast, certain extra-embryonic cells, such as syncytiotrophoblasts, remain non-ciliated [33]. Interestingly, human induced pluripotent stem cells (iPSCs) also exhibit primary cilia [55], highlighting their presence in undifferentiated cell states.

**FIGURE 1 ∣:**
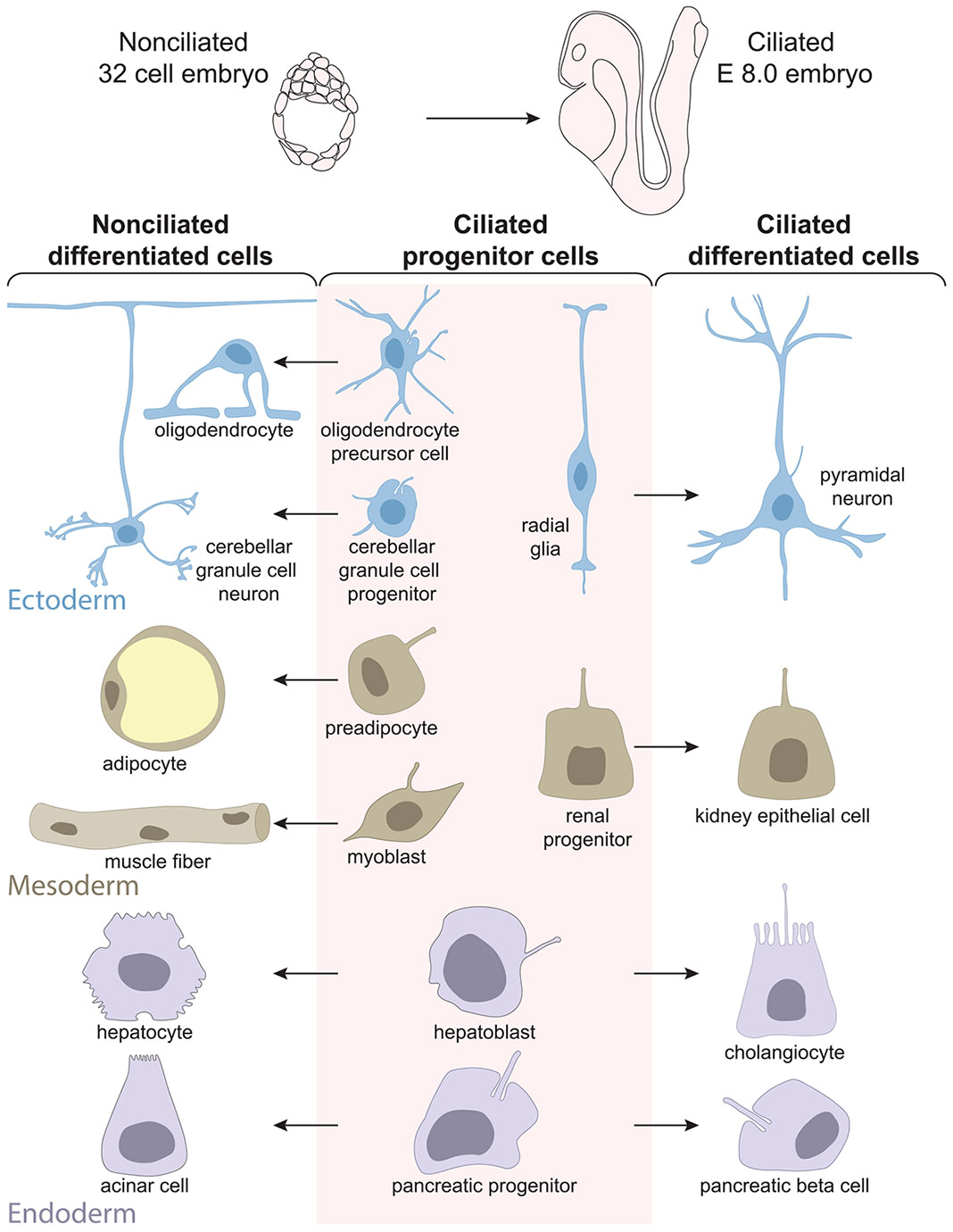
Both ciliated and non-ciliated cells derive from ciliated progenitor cells. Cells in early embryos lack primary cilia, but all non-dividing cells gain primary cilia during mouse development by E8.0 [33]. Ciliated progenitors in any dermal layer—ectoderm, mesoderm, or endoderm—can differentiate into ciliated or non-ciliated cells. Some progenitor populations give rise to both ciliated and non-ciliated cells.

The primary cilia present on progenitor cells persist during the differentiation of some cells, but are lost during the differentiation of others, as illustrated in Figure 1. What determines whether primary cilia are lost during differentiation? Cell type is not the primary determinant. For example, many types of cortical neurons are ciliated [24], while cerebellar GC neurons are not [22, 23]. Embryonic lineage does not determine ciliation state: both ciliated and non-ciliated cells arise from ectodermal, mesodermal, and endodermal progenitors. For example, preadipocytes and myoblasts from the mesoderm mature into non-ciliated cells while nephron progenitor cells in the same layer give rise to ciliated kidney epithelial cells [56]. Individual progenitors can also give rise to both ciliated and non-ciliated cells, as exemplified in Figure 1 by the hepatoblast, which can differentiate into a non-ciliated hepatocyte or a ciliated cholangiocyte [26, 27], and pancreatic precursor cells, which can differentiate into non-ciliated exocrine acinar cells or ciliated alpha, beta or delta islet cells [42]. Primary cilia loss during differentiation appears to be determined by the cellular response to differentiation cues.

The timing of primary cilia loss differs across different developmental pathways. Loss of primary cilia can occur during terminal differentiation, such as in cerebellar GC progenitors [22, 23] or adipocytes [28, 29], or it can happen at earlier stages. Ciliated oligodendrocyte precursor cells differentiate first into intermediate pre-myelinating oligodendrocytes before they become oligodendrocytes, and neither cell is ciliated [24, 25]. Hematopoietic precursor cells are also ciliated [57], but they give rise to unciliated lymphoid and myeloid progenitor cells, which further differentiate into diverse non-ciliated blood cells [16]. Ciliation state can also change during multiciliated cell differentiation, which progresses through monociliated intermediates [7, 58]. In all these cases, of primary cilia loss appears coordinated with the cellular differentiation program.

## 4 ∣ Switching Between Ciliated and Non-Ciliated States

**FIGURE 2 ∣:**
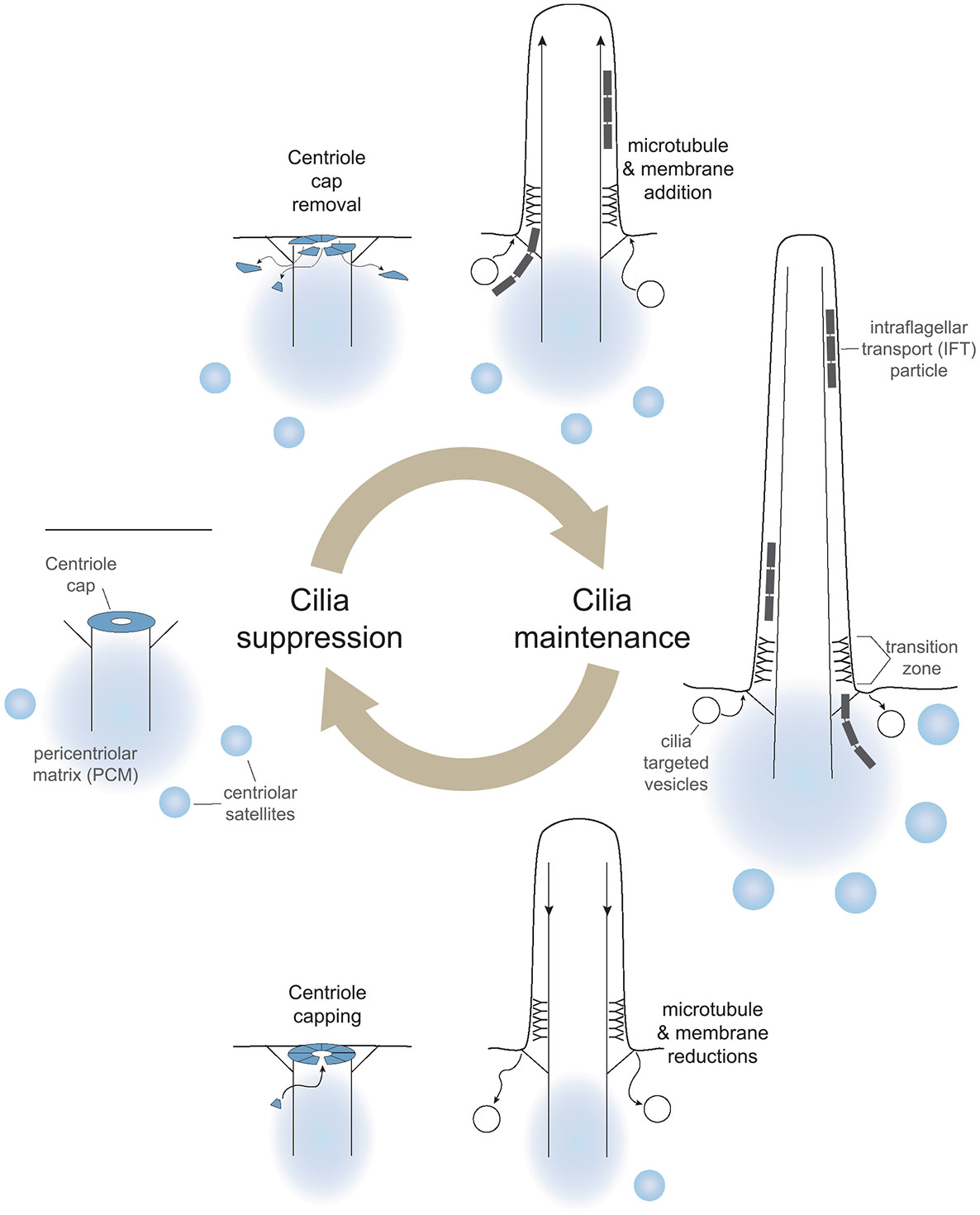
The ciliation state of a cell can be altered by ciliogenesis or primary cilia disassembly. The centriole cap complex (blue) assembled on the distal end of the mother centriole suppresses primary cilia growth. Ciliogenesis involves both cap removal and extension of ciliary microtubules and membranes. Once established, primary cilia are maintained by continual ciliary component delivery and retrieval. Primary cilia disassembly involves axoneme microtubule depolymerization and, eventually, mother centriole recapping. Centriolar satellites and the pericentriolar matrix both support the generation and maintenance of primary cilia.

How is the presence of primary cilia in progenitor cells connected to the persistence or loss of cilia in differentiated cells? Because primary cilia disassemble each time a cell divides [59], the assumption has been that primary cilium absence in differentiated cells is the result of failed cilia reformation after mitosis. The absence of primary cilia was thought to be a default state. Emerging evidence, however, including the presence of primary cilia on post-mitotic cells that become non-ciliated, suggests that there may be multiple ways cells accomplish primary cilia loss. In addition, there are capping complexes that can actively prevent ciliogenesis [60]. As illustrated in Figure 2, conversion between ciliation states involves both remodeling the distal end of the mother centriole and primary cilia growth or disassembly. In this section, we examine the important processes that suppress, build, and maintain primary cilia. In the following section, we will delve into the different pathways of primary cilia loss in more detail.

### 4.1 ∣ Primary Cilia Suppression

Primary cilia are absent both on cells exiting the mitotic cycle and on many differentiated cells (Table 1). The absence of primary cilia is not a default state: it is established through centriolar cap complex binding to the distal end of the centriole. The primary components of this complex are the proteins CP110 and CEP97 [60]. CP110 can bind microtubule plus ends directly in vitro [61] and likely functions in vivo by preventing microtubule growth. CEP97 recruitment is dependent on the centriolar kinesin KIF24 and KIF24’s recruitment of M-Phase Phosphoprotein 9 (MPP9) [62, 63]. When centrioles duplicate in S-phase, the CP110/CEP97 complex is involved in determining centriole length and remains in place which suppresses premature cilia growth [64]. In vitro, little or no CP110/CEP97 bound to microtubule plus ends undergoes turnover [61]. Although reduced centriolar cap recruitment generally promotes ciliogenesis [60, 62, 63, 65], experiments in mouse and *Xenopus* show that loss of CP110 can impair primary cilia formation, suggesting it may also support ciliogenesis [66, 67].

Primary cilia suppression in cerebellar GC neurons prevents regrowth after disassembly. During neurogenesis, mother centriole docking coincides with primary cilia loss [22]. Though docking can initiate ciliogenesis, we recently found that mother centriole capping increased as primary cilia frequency decreased. In adult GC neurons, which lacked primary cilia but had docked mother centrioles, capping was nearly 100%. We concluded that centriole cap binding prevents docked mother centrioles from reforming cilia [68]. Docking without ciliogenesis was unexpected; previously, the only known example of centriole docking without primary cilium extension was the transient docking of CP110-capped centrioles at the immune synapse [69].

### 4.2 ∣ Ciliogenesis

The initial membrane recruitment and axoneme microtubule extension during ciliogenesis is coordinated with the removal of the CP110/CEP97 complex from the distal mother centriole (reviewed in Zhao et al. [70]). Potential mechanisms for cap removal involve eventual CP110 degradation after dissociation from the centriole (reviewed in Xie et al. [71]). Centriole cap reformation might be prevented by continuous suppression of CP110 translation by the microRNA, miR-1239-3p [65].

There are two ciliogenesis pathways that differ in basal body position and ciliary membrane recruitment: the intracellular and extracellular pathways. Here, we highlight the essential elements of these processes (reviewed in Breslow and Holland [59]). Mother centrioles far from the cell cortex use the intracellular pathway, involving centrosome cap removal and pal vesicle recruitment to the distal appendages. Membrane addition continues as the microtubules grow to form the axoneme. The primary cilium origin remains recessed from the cell surface, and the ensheathing plasma membrane is referred to as the ciliary pocket [72, 73]. The extracellular pathway generates primary cilia that emerge directly from the cell surface (this example was utilized in Figure 2). Here, centriole cap removal is coordinated with mother centriole docking at the plasma membrane rather than vesicle recruitment [59].

Many primary cilia components move via IFT trains, using kinesin-II to move toward the primary cilia tip and dynein 2 to return to the base [9]. Deletion of cilia-specific motors or IFT components is embryonic lethal in mammals [17, 18, 50, 74]. Cellular structures outside the cilium are important for the synthesis/assembly and recruitment of cilia components. The pericentrosomal material (PCM) surrounds the mother centriole [75] and supports primary cilium growth [76]. Centriolar satellites, which are non-membranous organelles typically congregated near the centrosome, are also needed for ciliogenesis [77, 78], though how the structures work in concert is unclear. Primary cilia and centrosomal proteins can be synthesized at the centriolar satellites [79], which then partition into the PCM to facilitate entry into the primary cilium.

Vesicle-mediated delivery of membrane is essential for the growth of all cilia. RAB8- and RAB11-positive endosomes deliver both the elements of the lipid bilayer and many of the transmembrane proteins. ARF family GTPases also regulate ciliary membrane cargo delivery, important for ciliogenesis, cilia maintenance, and cilia function [70].

### 4.3 ∣ Primary Cilia Maintenance

Primary cilium presence on a cell reflects more than just the result of successful completion of ciliogenesis. Cilia are dynamic structures, and without continuous upkeep, they are prone to disassembly [76]. What does it take to maintain a primary cilium? Although requirements may vary between cell types, several core features must be preserved to prevent primary cilia loss: (1) the length of the axoneme microtubules [80]; (2) the balance of membrane delivery and retrieval [81, 82]; (3) lipid composition [10]; (4) the selectivity barrier [8]; (5) the rate of component turnover [2]; (6) lack of triggers of disassembly [83]; and (7) the cellular structures supporting cilia including the PCM [84], centriolar satellites [85], and the cytoskeleton [86].

The requirements for primary cilia formation, function and maintenance overlap [76]. As axoneme microtubules grow, they are stabilized by both post-translational modifications and microtubule binding proteins [87]. The role of IFT in cilia maintenance was demonstrated in *Chlamydomonas* flagella using temperature sensitive kinesin-II mutants that shorten upon inhibition of anterograde IFT [88] and in cultured fibroblasts by acute inhibition of kinesin-II that lose primary cilia [89]. Although axoneme microtubules do not treadmill like other microtubules in the cell, delivery of tubulin monomers via IFT, along with diffusion [90], contributes to cilia regeneration and maintenance in *Chlamydomonas* and *C. elegans* sensory neurons [91, 92]. Centriolar satellites [79, 85] and the PCM [84] are thought to contribute by facilitating the synthesis and trafficking of soluble cilia components during both ciliogenesis and cilia maintenance.

Other proteins outside the cilium contribute to primary cilia maintenance, including the tumor suppressor, von Hippel-Lindau protein, and GSK3*β* [93]. They could work in part by preventing extensive actin polymerization, which can reduce cilia length and frequency [94, 95]. The complex relationships between primary cilia and actin are reviewed in more detail in Mirvis et al. [86]. Another cytoskeletal component, the ciliary rootlet, can contribute to cilia maintenance in photoreceptor (and potentially other) cells [96].

## 5 ∣ Multiple Pathways for Cilia Disassembly

**FIGURE 3 ∣:**
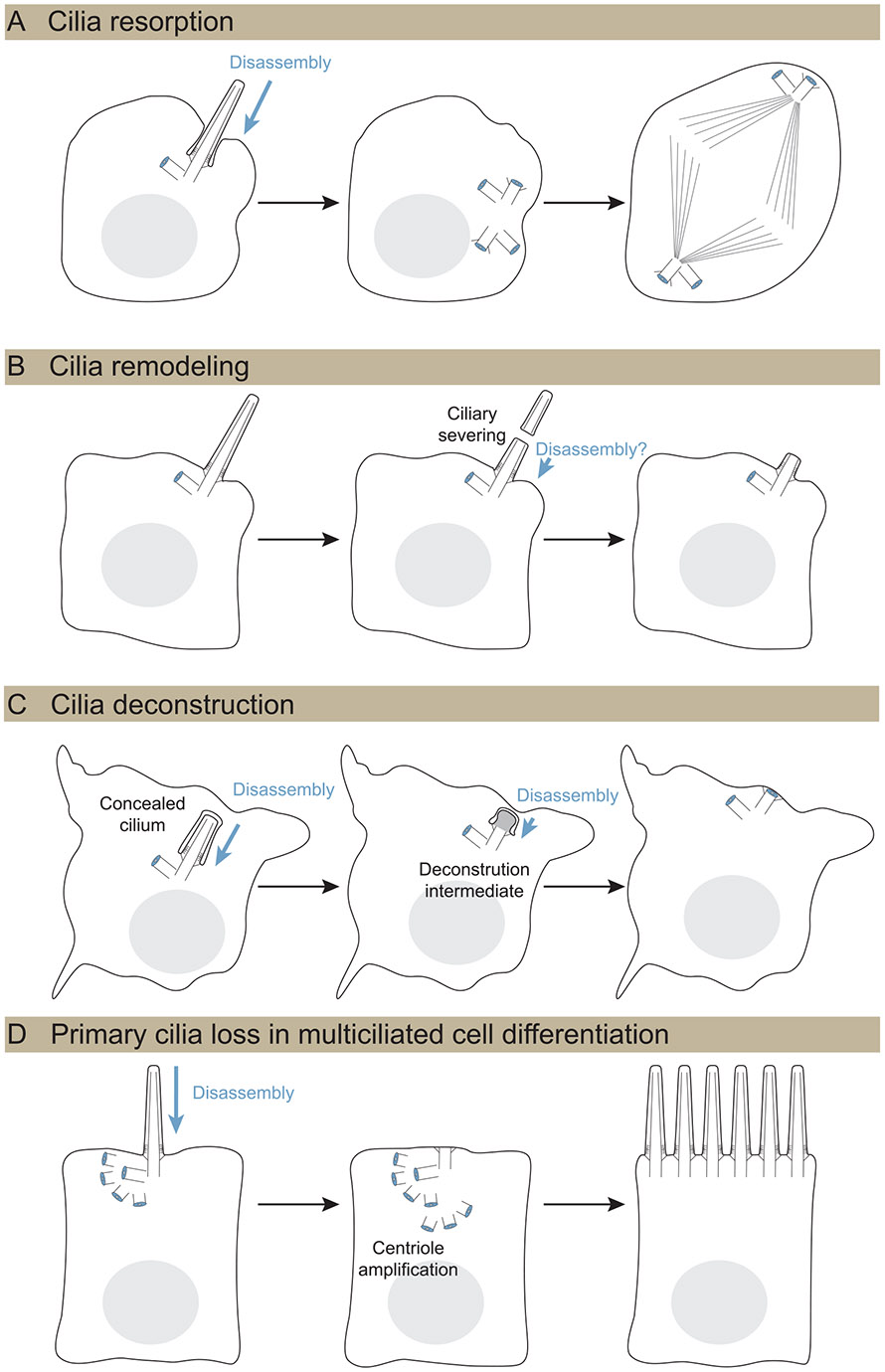
Primary cilia loss occurs through multiple pathways. (A) Primary cilia resorption is triggered by cell cycle kinases prior to mitosis. (B) Primary cilia remodeling acutely reduces the size of the primary cilium. Ciliary shortening occurs by microtubule destabilization and severing by the microtubule-severing enzymes, spastin, or katanin. Additional disassembly may be necessary after scission. (C) Cilia deconstruction involves the gradual withdrawal of components needed for primary cilia maintenance. Intracellular cilia undergo disassembly through deconstruction intermediates, followed by capping of the mother centriole, preventing reciliation. (D) Multiciliated cells (MCCs) have primary cilia early in differentiation that are disassembled prior to the formation of the secondary cilia. Centriolar cap complexes are marked in blue.

Primary cilia loss has been observed at the G1-S phase transition [12, 97], and until recently, this has been the primary model for studying the mechanisms of primary cilia loss [10]. The absence of primary cilia in the differentiated cells (Table 1) might result from suppressed ciliogenesis following pre-mitotic cilia resorption. However, there are additional primary cilia disassembly strategies, described below and illustrated in Figure 3, that are also utilized during differentiation. We include a description of primary cilia severing, which, although does not completely remove primary cilia, may serve as an initial step in ciliary remodeling.

### 5.1 ∣ Pre-Mitotic Primary Cilia Resorption

Cell cycle kinases trigger signaling cascades that drive key steps of cell division, including centriole duplication, which coincides with DNA replication in S-phase. Before mitosis begins in mammalian cells, kinases also trigger cilia disassembly. A prominent cell cycle kinase, Aurora kinase A (AURA), mediates histone deacetylase 6 (HDAC6) phosphorylation, which in turn promotes tubulin deacetylation [11]. AURA activation or stabilization is mediated by multiple factors, including human enhancer of filamentation 1, Pitchfork, Ca^2+^-CAM, Dishevelled 2, Polo-like kinase 1 (PLK1) complex, and trichoplein. In addition, phosphorylation of the microtubule depolymerizing kinesins KIF24 and KIF2A contributes to axoneme depolymerization (reviewed in Liang et al. [83]). Primary cilia loss also occurs during meiosis [98], but studies are needed to investigate if similar kinase regulatory strategies facilitate disassembly in those cells.

During disassembly, the microtubules are not the only component that must be considered. Ciliary membrane can be shed through ectosome release following serum stimulation of quiescent cultured cells [81, 82]. An early feature of such ciliary membrane remodeling in cultured cells involves the displacement of the primary cilia-enriched phosphoinositide 5’phosphatase, Inpp5e, from primary cilia, coupled with actin polymerization in cilia, and decapitation of primary cilia tips [81, 82].

It is unclear why cells are non-ciliated during mitosis. Although common in mammals, there are organisms that retain primary cilia as they divide (see Riparbelli et al. [99] and references therein). A remnant of the cilia membrane has been found to remain with the mother centriole of neural progenitors in the developing neocortex [100], but many mitotic cells completely resorb primary cilia. Perhaps once committed, mammalian cells become insensitive to signals that might alter cellular programming. Primary cilia might also restrict centriole movement preventing transit to opposing poles. In fact, in meiosis, the primary cilium is thought to contribute mechanical stability for the zygotene chromosomal bouquet [98]. Many proteins involved in ciliogenesis and primary cilia maintenance also participate in midbody formation and abscission [101, 102], so primary cilia persistence could decrease cytokinesis efficiency.

### 5.2 ∣ Cilia Remodeling

Cilia remodeling is prevalent in multiple differentiated tissue contexts in both primary cilia and multicilia. As neurons in the developing chick and mouse neural tube undergo delamination by abscission from the ventricles, primary cilia rapidly shorten, leaving only an ARL13B+ particle [103, 104]. This short primary cilium grows longer during apical process retraction [103]. Choroid plexus cells are multiciliated, but their cilia lack the central microtubules and other apparatus required for motility and instead more closely resemble the microtubule configuration of primary cilia. Cilia on these cells shorten late in embryogenesis through a microtubule destabilization process controlled by deglutamylation and the microtubule-severing enzyme, spastin [105].

Primary cilia remodeling in differentiated cells has been connected to alterations in Sonic hedgehog (SHH) signaling, a pathway known to signal extensively through primary cilia [5, 106]. The remodeled primary cilium in neuroepithelial cells is essential for nascent axon establishment and maintenance [103]. The levels of GPR161, a negative regulator of the SHH pathway, increased as the neurons retracted from the surface, suggesting that cilia remodeling contributes to SHH pathway suppression [103]. In the choroid plexus, cilia remodeling attenuates SHH signaling and is important to prevent hydrocephalus [105, 107].

Rapid primary cilia shedding can be triggered by stress conditions or pharmacological induction [83]. Such deciliation can also be more prevalent than primary cilia resorption in some cultured cells [108]. The microtubule-severing enzyme katanin has been proposed to function in such severing [109], and is coupled with basal body release before mitosis in *Chlamydomonas* [110]. In cultured kidney epithelial cells, katanin overexpression promotes primary cilia shedding [108]. As mentioned previously, primary cilia remodeling only shortens cilia; however, it could be coupled with other mechanisms to accomplish complete disassembly.

### 5.3 ∣ Primary Cilia Deconstruction

Although many cerebral neurons have primary cilia, the most abundant cerebellar neurons, GC neurons, lack primary cilia [22, 23]. However, both GC progenitors and early differentiating, post-mitotic GCs are ciliated [22]. While GC progenitor primary cilia undergo pre-mitotic cilia resorption after S-phase, both in culture and in vivo [22, 111], it seemed unlikely that postmitotic cells upregulate cell cycle kinases. Using volume EM, we investigated ultrastructural changes in primary cilia as GCs proliferate and differentiate in developing tissue. Unexpectedly, we found that many of the primary cilia in both progenitor and differentiating GCs were completely internal, concealed within the cells. As differentiation progressed, we found novel primary cilia disassembly intermediates, and, in the most mature cells, mother centrioles docked at the plasma membrane that lacked primary cilia [22]. Transcriptomic and immunofluorescent analysis revealed that the gradual deconstruction coincides with decreased abundance of centriolar satellite, PCM, and IFT components [68]. These results suggested a new mechanism for primary cilia disassembly—deconstruction caused by withdrawal of cilia maintenance. Although cilia maintenance was known to be important for disease prevention [76], withdrawal of maintenance had not previously been considered a plausible mechanism for primary cilia disassembly.

Primary cilia deconstruction and resorption are both coupled to other cellular programs but differ in several ways. Pre-mitotic resorption in cultured cells rapidly reduces cilia frequency within two hours [11, 108], while primary cilia deconstruction during GC migration and differentiation is more gradual, though the exact duration is unknown. Only primary cilia resorption is actively triggered; AURA and PLK1, which regulate cell cycle re-entry, are transcriptionally downregulated in the postmitotic differentiating GC neurons [68]. In contrast to kinase-activated signaling mechanisms, loss of cilia maintenance is cumulative, which could contribute to heterogeneity in deconstruction [22]. Volume EM shows unique disassembly intermediates including some with internal vesicles, a subset that were bulbous with denser cilioplasm and late-stage intermediates with only intracellular ciliary vesicles anchored to the centriole distal appendages. Similar intermediates might exist during ciliogenesis but are rarely observed [112]. Some deconstruction intermediates resembled early membrane recruitment to the mother centriole during intracellular ciliogenesis. Although distinct from pre-mitotic resorption, primary cilia deconstruction effectively removes cilia from the large population of GC neurons. Moreover, reduced maintenance and mother centriole recapping together prevent re-ciliation in GC neurons [68].

Does primary cilia deconstruction happen outside the cerebellum? We think it might occur during post-mitotic differentiation of some of the cells listed in Table 1. For example, keratinocytes in the skin’s granular layer are post-mitotic, non-ciliated cells that differentiate from ciliated progenitor cells in the basal layer [19]. Ultrastructural studies revealed that, like during GC differentiation, cells transitioning from the basal layer to the spinous layer exhibited progressively shorter, intracellular ciliary intermediates [20]. Hopefully, awareness of the prevalence of non-ciliated cells will lead to further investigations to determine the cilia disassembly pathways occurring in non-dividing cells.

### 5.4 ∣ Primary Cilia Disassembly in Differentiating Multi-Ciliated Cells

Multiciliated cells are found on airway epithelial cells, ependymal cells in the brain ventricles, and lining the oviduct. As these cells differentiate, a primary cilium forms and is then disassembled prior to multiciliogenesis [7]. The antecedence of primary cilia was later confirmed by multiple groups who showed primary cilia are gone before motile cilia emerge using immunofluorescence with markers that distinguish between the two [113, 41]. In mouse ependymal progenitor cells, primary cilia are present when centriole amplification starts [114]. In these cells, the daughter centriole can also mature, and a second primary cilium can form; however, both disassemble prior to multiciliogenesis [58].

Primary cilia disassembly during multiciliated cell differentiation has never been mechanistically explored. Because deconstruction involves depletion of ciliary components, that pathway is not likely to cause primary cilia loss in these cells, which are generating an abundance of cilia components in preparation for multiciliogenesis. Recent evidence has revealed that multiciliated cells utilize components of the cell cycle during differentiation [115-117]. Perhaps primary cilia loss prior to multicilia growth utilizes cell cycle kinases, as in pre-mitotic cilia resorption. It is well established that parental centrioles are not required for multiciliogenesis in culture [58, 118, 119]. However, the importance of signals detected by primary cilia or the consequence of disrupting disassembly during multiciliogenesis in vivo has not been investigated.

An interesting variation happens in specialized multiciliated olfactory sensory neurons (OSNs). In mammals, olfactory receptors localize to OSN cilia, located at the end of the dendritic knobs. These neurons start as mono-ciliated progenitors embedded in the olfactory epithelium. Later, multiple centrioles form in addition to the basal body, and the primary cilium is resorbed. Next, the dendritic endings of the receptor cells increase in width and begin to sprout long multicilia [120, 121]. Centriole amplification in olfactory neuron progenitors precedes at least one round of cell division [122, 123]. Unlike airway epithelial cells that do not proliferate during differentiation, the resorption of primary cilia noted in OSNs [120, 121] might utilize the pre-mitotic resorption pathway.

## 6 ∣ Altered Ciliation States Can Contribute to Pathogenesis

Ciliopathies can be caused by mutations that reduce ciliogenesis [10]. Are other diseases impacted by changes in ciliation state? If we ask the question, are tumor cells ciliated, the answer—that some are and some are not [124]—provides little insight. But if we consider that some tumors originate from ciliated cells and others from non-ciliated cells, we might instead ask which tumors have a different ciliation state than healthy cells? Can ciliation of non-ciliated cells caused by failure to suppress primary cilia or disruptions in primary cilia disassembly contribute to pathology? Or can failures in primary cilia maintenance regulate disease progression? The following examples of pathological conditions involving aberrant ciliation states suggest that rethinking the role of ciliation state in disease may lead to new insights and possibly novel pharmacologic targets.

### 6.1 ∣ Primary Cilia Maintenance Defects Contribute to Cilia Loss in Melanoma

Melanocytes are ciliated cells that produce pigment in the skin. Melanocyte proliferation can result in benign nevi or malignant melanoma. Whereas nevi cells remain ciliated, the melanoma cells have low primary cilia counts. Loss of primary cilia in melanoma cells is likely independent of proliferation and cell cycle progression [19]. *EZH2*, the G1-S transition-regulating transcription factor, might be working independent of the cell cycle to downregulate cilia-related genes [125]. Reversion back to a ciliated state by inactivation of *EZH2* can restrict melanoma growth [125]. Thus, in the case of melanoma, cilia maintenance restricts cell proliferation.

### 6.2 ∣ Loss of Primary Cilia Suppression Leads to Cilia Regrowth in Pancreatic Ductal Adenocarcinoma

In the pancreas, exocrine acinar cells are unciliated, while many ductal cells have a primary cilium [42]. Interestingly, both contribute to pancreatic ductal adenocarcinoma (PDAC). Initial pancreatic ductal metaplasia can be caused by ciliary component depletion in the pancreatic epithelium [126], and PDAC cells are devoid of primary cilia [127]. However, a subpopulation of PDAC cells that stopped suppressing primary cilia formation was resistant to chemotherapy [128]. Primary cilia presence correlates with PDAC prognosis, and patients whose cancers were primary cilia-positive had a higher frequency of lymph node metastasis [129]. Failing to suppress ciliogenesis in PDAC cells results partly from melanophilin expression, which promotes ciliogenesis through unknown mechanisms and upregulates phospholipase C, promoting metastasis [130]. Although the role of cilia in PDAC is complex, evidence indicates that primary cilia state changes during disease progression lead to undesirable outcomes.

### 6.3 ∣ Primary Cilia Are Maintained Instead of Disassembled in SHH Medulloblastoma

Medulloblastoma (MB) in the cerebellum is the most common malignant childhood brain tumor. There is a subtype largely responsible for MB in children under 3 years that arises through unregulated GC neuron progenitor proliferation in response to SHH [131]. As mentioned previously, primary cilia deconstruction occurs as GCs differentiate. SHH MBs, however, are ciliated [132]. Persistent proliferative clusters of GC progenitor cells whose growth depends on primary cilia are present preceding SHH MB development [68, 133]. It seems likely that primary cilia are maintained in the progenitor cells contributing to tumorigenesis, or primary cilia resurgence contributes to reversion into progenitor-like cells, although further experiments are needed to test between these possibilities.

### 6.4 ∣ Primary Cilia Loss in Parkinson’s Disease

Many cerebral neurons and astrocytes are ciliated. However, primary cilia are lost in astrocytes and a subset of neurons in the striatum in people with autosomal dominant Parkinson’s disease caused by activating mutations in leucine-rich repeat kinase 2 (LRRK2) [46, 49, 134]. Both *Lrrk2* mutant mice and postmortem human brain tissue from Parkinson’s patients have cilia loss [46, 134]. Primary cilia loss in these striatal cells correlates with decreased secretion of glial-derived neurotrophic factor (GDNF) and, as a result, decreased neuroprotection for dopaminergic neurons [46, 134]. LRRK2 can phosphorylate many Rab GTPases, including RAB8A and RAB10, which are involved in trafficking to cilia [49]. In cultured cells, LRRK2 mutations can suppress ciliogenesis by preventing centriole cap removal and promote pre-mitotic cilia resorption [49].

The observed reductions in ciliation could be caused by activation of primary cilia disassembly or suppression of ciliogenesis. However, it is not yet clear what causes changes in the ciliation state of adult tissue. Parkinson’s patients live for many years before symptoms emerge. Although many astrocytes retain proliferative potential, neurons are post-mitotic cells. Therefore, the ability of LRRK2 mutations to suppress ciliogenesis and promote premitotic resorption in culture may not be physiologically relevant to the cilia loss observed in Parkinson’s patient neurons. If ciliogenesis occurs normally during neurogenesis, it is possible that aberrant LRRK2 activity promotes primary cilia deconstruction or another mode of cilia disassembly.

## 7 ∣ Conclusions

Primary cilia absence has largely been considered a pathogenic consequence of failed ciliogenesis. However, non-ciliated cells are abundant, and interestingly, most derive from cell lineages that include ciliated cells. Here, we described several pathways of primary cilia disassembly in differentiated cells that need mechanistic investigation. In addition, open questions remain as to which non-ciliated cells arise from primary cilia suppression after mitosis, and which utilize cilia disassembly pathways in post-mitotic cells. It is currently unclear how cilia remodeling contributes to cilia loss. What are the benefits of primary cilia absence in certain tissues? We need further studies to understand how cilia suppression and maintenance regulate signal perception in differentiated cells. Additional studies are also needed to investigate how changes in ciliation contribution to disease. How might dysregulated primary cilia suppression and maintenance contribute to reversion into progenitor/stem cells and tumorigenesis? Could these pathways be exploited therapeutically?

Many transitions within cell lineages from ciliated to non-ciliated states occur in complex tissues, as does the pathogenic persistence of cilia and the reappearance of cilia in cells that are normally non-ciliated. To gain a deeper understanding, experiments outside cultured cell systems may be required. In addition to volume EM, super-resolution, and expansion microscopy will make addressing these questions more tractable than ever before. In the future, intravital imaging and mouse models expressing fluorescent cilia and cell cycle biosensors [135] could make it possible to discriminate between cilia disassembly pathways by monitoring primary cilia status in developing tissues in vivo. We anticipate that investigations into how, when, and where transitions between ciliation states occur will reveal important molecular mechanisms and new insights into how both the presence and absence of primary cilia affect disease progression.

## Figures and Tables

**TABLE 1 ∣:** Differentiated, non-ciliated cells.

Oligodendrocytes and pre-myelinating oligodendrocytes	[24, 25]
Microglia	[24]
Cerebellar granule cell neurons	[22, 23]
Hepatocytes	[26, 27]
Adipocytes	[28, 29]
Retinal pigment epithelial cells	[30]
Luminal mammary epithelial cells	[31, 32]
Myotubes	[21]
Placenta syncytiotrophoblasts	[33]
Steroidogenic adrenal cortical cells	[34, 35]
Pulmonary endothelial cells	[36]
Stomach/gut epithelia	[37]
Esophagus epithelial cells	[38]
Kidney podocytes	[39]
Intercalated cells of the kidney collecting duct	[40]
Squamous epithelial cells in lung alveoli	[37]
Lung Clara cells (secretory epithelial cells)	[41]
Blood cells, both myeloid and lymphoid lineages	[16]
Pancreatic exocrine acinar cells	[42]
Osteoclasts	[43]
Adult keratinocytes, granular layer	[19, 20]
Cochlear inner hair cells	[44]

## Data Availability

Data sharing is not applicable to this article because no datasets were generated or analyzed for the Problems and Paradigms BioEssay.
